# Filling the psycho-social gap in the EQ-5D: the empirical support for four bolt-on dimensions

**DOI:** 10.1007/s11136-020-02576-5

**Published:** 2020-07-09

**Authors:** Gang Chen, Jan Abel Olsen

**Affiliations:** 1 Centre for Health Economics, Monash Business School, Monash University, Victoria, 3145 Australia; 2 Department of Community Medicine, University of Tromsø, 9037 Tromsø, Norway; 3 Division of Health Services, Norwegian Institute of Public Health, 0213 Oslo, Norway

**Keywords:** Health-related quality of life, Life satisfaction, Health utility, EQ-5D, Bolt-on

## Abstract

**Purpose:**

The EQ-5D is the most widely applied generic preference-based measure (GPBM) of health-related quality of life (HRQoL). Much concern has been raised that its descriptive system is lacking psycho-social dimensions. A recent paper in this journal provided theoretical support for four dimensions to fill this gap. The current paper aims to provide empirical support for these suggested bolt-on dimensions to the EQ-5D.

**Methods:**

We use data from the comprehensive Multi-Instrument-Comparison (MIC) study. The four proposed bolt-on dimensions (Vitality, Sleep, Social Relationships, and Community Connectedness) were selected from the Assessment of Quality of Life (AQoL)-8D. We investigate the relative importance of these four dimensions as compared to the five EQ-5D-5L dimensions on explaining HRQoL (measured by a visual analogue scale; *N* = 7846) or global life satisfaction (measured by the Satisfaction With Life Scale; *N* = 8005), using the Shorrocks-Shapely decomposition analysis. Robustness analyses on Vitality was conducted using data from the United States National Health Measurement Study (NHMS) (*N* = 3812).

**Results:**

All five EQ-5D-5L dimensions and four bolt-on dimensions significantly explained the variance of HRQoL. Among them, Vitality was found to be the most important dimension with regard to the HRQoL (relative contribution based on the Shorrocks-Shapely decomposition of *R*^2^: 23.0%), followed by Usual Activities (15.1%). Self-Care was the least important dimension (relative contribution: 5.4%). As a comparison, when explaining global life satisfaction, Social Relationships was the most important dimension (relative contribution: 24.0%), followed by Anxiety/Depression (23.2%), while Self-Care remained the least important (relative contribution: 1.6%). The importance of the Vitality dimension in explaining HRQoL was supported in the robustness analysis using the NHMS data (relative contribution: 23.7%).

**Conclusions:**

We provide empirical support for complementing the current EQ-5D-5L descriptive system with a coherent set of four bolt-on dimensions that will fill its psycho-social gap. Such an extended health state classification system would in particular be relevant for programme evaluations within the expanding fields of mental health and community care.

**Electronic supplementary material:**

The online version of this article (10.1007/s11136-020-02576-5) contains supplementary material, which is available to authorized users.

## Introduction

The EQ-5D is the most widely applied generic preference-based measure (GPBM) of health-related quality of life (HRQoL) [1]. Its original descriptive system has now been extended in terms of levels from the three level version (EQ-5D-3L) to the five level version (EQ-5D-5L) [2]. The EQ-5D has gained immense policy impact in several countries by being the recommended GPBM for use in economic evaluations that are to inform health service decision-making.

Following the increased policy attention on mental health, and the consequences of ill health on social isolation, concerns have been raised that the EQ-5D dimensional structure does not sufficiently include mental and social aspects of health. A solution would be to extend the descriptive system by adding ‘bolt-on’ dimensions [3]. Thus, for programme evaluations within wider fields of mental health and community care, we argue that the existing EQ-5D should be complemented by an additional psycho-social part consisting of four bolt-on dimensions. Such an extended descriptive system would be more aligned with the seminal WHO definition that emphasises three key dimensions of health: physical, mental, and social [4].

**Fig. 1:**
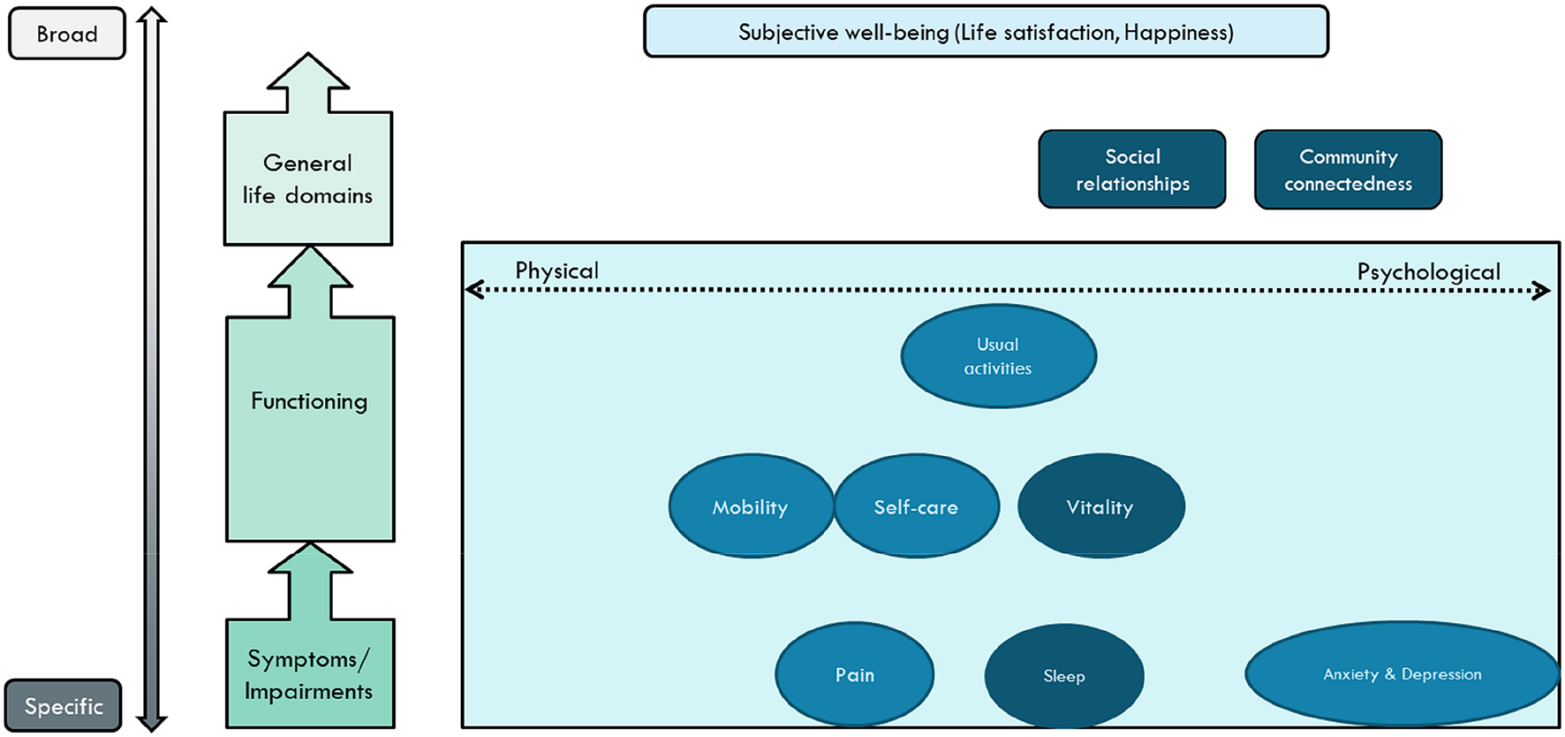
EQ-5D dimensions plus four bolt-on dimensions as set within a conceptual map

The aim of the current paper is to inquire into the empirical support behind four bolt-on dimensions to the EQ-5D that were recently suggested by Olsen and Misajon (O&M) in this journal [5]. The chosen bolt-ons result from their analytical approach to identify common denominators in the existing GPBMs, including the new PROMIS instrument [6], and to consider relevant dimensions in the Personal Well-being Index [7]. The pattern of identified dimensions was visualised within a conceptual map that focused on a < cause – effect > axis and a < physical – mental > axis. Figure 1 illustrates where the EQ-5D dimensions, together with the four additional psycho-social dimensions, would fit into this conceptual map.

The reasoning behind the suggested bolt-ons are as follows: *Vitality,* because it is included in four other GPBMs, though appearing with synonymous concepts such as energy or fatigue. And vitality connotes to the increasingly prevalent symptoms of fatigue. *Sleep,* because it is included in three other instruments. And sleep problems may be caused by underlying symptoms of nervousness and distress, and, not least; sleep is a word that does not require further explanations. *Social Relationships* capture social functioning with the inner circle of family and friends, and *Community Connectedness* measure the degree of social isolation. See O&M [5] for more theoretical support of these four bolt-on dimensions.

The current paper is structured as follows: Next section presents the data and the methods. The results section focuses on the relative importance of the nine dimensions for explaining variations in HRQoL and global life satisfaction (GLS, one component of subjective wellbeing). Lastly, the discussion section demonstrates the potential performance of the proposed four bolt-on dimensions, and points to some important areas for further research.

## Method

We use data from the Multi-Instrument-Comparison (MIC) study, which represents the world’s largest available data set to compare existing HRQoL and subjective well-being [8–10]. In addition to the five EQ-5D-5L dimensions included in MIC, the four bolt-ons proposed by O&M were drawn from the Assessment of Quality of Life (AQoL)-8D instrument [11]. Similar to the EQ-5D-5L, each of these four bolt-ons are described using five levels in AQoL-8D except for *Social Relationships* (which original has 6 levels but the responses from the bottom two levels were collapsed in this study). See Table 1 for the detailed wordings of these items.

**Table 1:** Four ‘bolt-on’ dimensions from the Assessment of Quality of Life (AQoL)-8D

Vitality
How much energy do you have to do the things you want to do? I am [Q1]
Always full of energy
Usually full of energy
Occasionally energetic
Usually tired and lacking energy
Always tired and lacking energy
Sleep
How often do you have trouble sleeping? [Q12]
Never
Almost never
Sometimes
Often
All the time
Social relationships (family & friends)
How satisfying are your close relationships (family and friends)? [Q10]
Very satisfying
Satisfying
Neither satisfying nor dissatisfying
Dissatisfying
Unpleasant/very unpleasant^a^
Community connectedness
How often do you feel socially isolated? [Q31]
Never
Rarely
Sometimes
Often
Always

The original question number (Q#) of AQoL-8D instrument is listed in brackets

^a^The Social Relationships dimension is described using six levels in AQoL-8D, but for the purpose of consistent analysis here, the bottom two levels were collapsed into one

In addition, the data from United States National Health Measurement Study (NHMS) [12] were used to confirm the finding that *Vitality* turned out to be so important in our MIC-based analyses. Similar to the MIC, the main purpose of NHMS was to compare commonly used GPBM based on a cross-sectional survey conducted among adults in the USA. Different from the MIC study, the 3L version of the EQ-5D was used in NHMS. Furthermore, as the NHMS did not include the AQoL-8D, we were not able to test all four bolt-ons considered in the main analysis. The most important bolt-on been identified (i.e. *Vitality*) was drawn from the corresponding dimension in the SF-6D in this robustness analysis. In addition, only EQ-VAS is available in NHMS data, but not the GLS indicator.

The statistical analyses seek to identify correlations across the nine dimensions and to show the contributions that the four bolt-on dimensions have for explaining variations in health and well-being, respectively, using the Shorrocks-Shapely decomposition analysis (with regard to the R-squared statistics post the ordinary least squares regression) [13, 14]. Health was measured using a visual analogue scale (VAS) (0–100 represented death and best possible health (physical, mental, social)), while global life satisfaction was measured by the first three items of Satisfaction With Life Scale (SWLS; Cronbach's alpha = 0.93, calculated based on the MIC data) [15, 16]. Since the raw scores of VAS and SWLS are on different scales, to facilitate a clear comparison on the regression coefficients, in the empirical analysis, both the raw scores of VAS and SWLS were rescaled onto a 0–1 score by using the formula: rescale score = (raw total score—theoretical minimum total score)/ (theoretical maximum total score—theoretical minimum total score). Take the 3-item SWLS score as an example, each item scores between 1 and 7 as such the rescale score was calculated as (raw total score-3)/(21–3). Finally, an exploratory factor analysis (EFA) was conducted using maximum likelihood method to show the latent structure of the original five EQ-5D-5L dimensions and the four bolt-ons. Given that all quality of life items are ordinal variables, the Spearman rank-order correlation was used to calculate the correlation matrix. The number of factors to be extracted was determined using the minimum average partial method, which has been found to outperform other methods [17, 18]. Rotation was performed using an oblique Promax method to allow for potential correlations among factors.

We also briefly demonstrated the potential performance of EQ-5D-5L plus the four bolt-on dimensions using a brief direct mapping analysis (based on the ordinary least squares estimator), in which the nine dimensions were mapped onto AQoL-8D utility scores. All nine dimensions were included in the regression analysis as a series of dummy variables to allow for the potential non-linear effect. The AQoL-8D was in particular developed to expand the psycho-social health dimensions of the GPBM. However, the comprehensiveness of the 35-item AQoL-8D classification system may potentially hinder its application in clinical trials or large-scale population surveys. In the Electronic supplementary material, we investigated to what extent that the EQ-5D-5L plus the four bolt-on dimensions can explain the variations of the AQoL-8D utility scores. In addition to the EFA, which was conducted using EViews version 11 (IHS Global Inc., Irvine, CA, USA), all other analyses were conducted using Stata version 14.1 (StataCorp LP, College Station, Texas, USA).

## Results

The MIC data include a total of 8022 respondents (48% male; 18% aged 18–34 years, 35% aged 35–54 years, 25% aged 55–64 years and 22% aged 65 and above). After excluding missing values on VAS or SWLS, we have 7846 respondents in the analyses of VAS values (mean raw score ± standard deviation: 67.1 ± 21.7) and 8,005 respondents in the analysis of SWLS values (mean raw score ± standard deviation: 13.3 ± 4.6). For detailed descriptive statistics, see Electronic Supplementary Material 1 and [8–10].

Table 2 presents the Spearman correlation coefficients among the nine HRQoL dimensions, the VAS, and the SWLS. Among the EQ-5D-5L dimensions, *Usual Activities* had the largest magnitude of correlations with VAS (*r* =  − 0.520), while *Anxiety/Depression* had the largest magnitude of correlation with SWLS (*r* =  − 0.530). When further considering the four bolt-on dimensions, the strongest correlation with VAS was found to be *Vitality* (*r* =  − 0.600). Overall, the four bolt-on dimensions had stronger correlations with *Anxiety/Depression* than did the other four EQ-5D-5L dimensions.

**Table 2:** Correlations among VAS, SWLS, and health-related quality of life domains

	VAS	Mobility	Self-care	Usual activities	Pain/discomfort	Anxiety/depression	Vitality	Sleep	Social relationships	Community connectedness	SWLS
VAS	1										
Mobility	− 0.449	1									
Self-care	− 0.336	0.519	1								
Usual activities	− 0.520	0.694	0.536	1							
Pain/discomfort	− 0.480	0.595	0.389	0.590	1						
Anxiety/depression	− 0.462	0.254	0.244	0.375	0.341	1					
Vitality	− 0.600	0.419	0.297	0.505	0.454	0.520	1				
Sleep	− 0.440	0.327	0.258	0.398	0.429	0.459	0.514	1			
Social relationships	− 0.368	0.166	0.189	0.258	0.205	0.463	0.403	0.322	1		
Community connectedness	− 0.407	0.272	0.262	0.353	0.290	0.532	0.474	0.403	0.506	1	
SWLS	0.536	− 0.235	− 0.207	− 0.318	− 0.283	− 0.530	− 0.487	− 0.385	− 0.514	− 0.481	1

All Spearmen's coefficients are statistically significant (all *P* < 0.01)

*SWLS* satisfaction with life scale; *VAS* visual analogue scale. The SWLS was calculated as a summary score of the first three items of the SWLS instrument and then rescaled onto the 0–1 scale. The VAS was originally recorded on a 0–100 scale and was rescaled onto 0–1 scale

To what extent each of these nine dimensions can explain the variance of VAS and SWLS are reported next. Among the nine dimensions (see Supplementary Table 3), *Vitality* and *Self-care* had the largest and smallest *R*^2^ (0.37 versus 0.15) for explaining VAS. When explaining SWLS, *Anxiety/Depression* had the largest *R*^2^ (0.31), while *Self-care* had the lowest (0.05).

**Table 3:** Experience weighting on EQ-5D plus bolt-on dimension(s)

	MIC data			NHMS data	
	(1)	(2)	(3)	(4)	(5)
	VAS	VAS	SWLS	EQ-VAS	EQ-VAS
Mobility					
Level 2	− 0.034***	− 0.026***	0.003	− 0.066***	− 0.051***
	(0.006)	(0.005)	(0.007)	(0.008)	(0.008)
Level 3	− 0.059***	− 0.045***	− 0.006	0.024	0.006
	(0.008)	(0.008)	(0.010)	(0.042)	(0.041)
Level 4	− 0.098***	− 0.082***	− 0.008		
	(0.013)	(0.012)	(0.015)		
Level 5	− 0.109***	− 0.092***	− 0.025		
	(0.033)	(0.031)	(0.039)		
Self-care					
Level 2	− 0.024***	− 0.022***	0.009	− 0.064***	− 0.051***
	(0.008)	(0.007)	(0.009)	(0.013)	(0.013)
Level 3	− 0.002	− 0.008	0.029**	− 0.097*	− 0.080*
	(0.012)	(0.011)	(0.014)	(0.050)	(0.049)
Level 4	− 0.014	− 0.013	0.087***		
	(0.023)	(0.022)	(0.027)		
Level 5	0.019	0.018	0.135*		
	(0.059)	(0.056)	(0.070)		
Usual activities					
Level 2	− 0.075***	− 0.047***	0.008	− 0.118***	− 0.092***
	(0.006)	(0.005)	(0.007)	(0.008)	(0.008)
Level 3	− 0.120***	− 0.077***	− 0.010	− 0.238***	− 0.193***
	(0.009)	(0.009)	(0.011)	(0.020)	(0.020)
Level 4	− 0.171***	− 0.116***	− 0.065***		
	(0.014)	(0.014)	(0.017)		
Level 5	− 0.165***	− 0.100***	− 0.006		
	(0.026)	(0.025)	(0.031)		
Pain/discomfort					
Level 2	− 0.041***	− 0.027***	0.000	− 0.055***	− 0.048***
	(0.005)	(0.004)	(0.006)	(0.006)	(0.006)
Level 3	− 0.079***	− 0.050***	− 0.009	− 0.148***	− 0.128***
	(0.006)	(0.006)	(0.008)	(0.015)	(0.015)
Level 4	− 0.104***	− 0.075***	− 0.006		
	(0.009)	(0.009)	(0.011)		
Level 5	− 0.147***	− 0.111***	− 0.012		
	(0.018)	(0.017)	(0.021)		
Anxiety/depression					
Level 2	− 0.061***	− 0.019***	− 0.074***	− 0.045***	− 0.025***
	(0.004)	(0.004)	(0.006)	(0.007)	(0.007)
Level 3	− 0.128***	− 0.042***	− 0.133***	− 0.127***	− 0.083***
	(0.006)	(0.006)	(0.008)	(0.020)	(0.020)
Level 4	− 0.213***	− 0.097***	− 0.168***		
	(0.009)	(0.010)	(0.012)		
Level 5	− 0.258***	− 0.116***	− 0.219***		
	(0.014)	(0.014)	(0.018)		
Vitality^a,b^					
Level 2		− 0.044***	− 0.031***		− 0.009
		(0.009)	(0.011)		(0.008)
Level 3		− 0.107***	− 0.069***		− 0.069***
		(0.009)	(0.011)		(0.009)
Level 4		− 0.168***	− 0.130***		− 0.106***
		(0.010)	(0.012)		(0.012)
Level 5		− 0.225***	− 0.147***		− 0.156***
		(0.012)	(0.015)		(0.014)
Sleep^a^					
Level 2		− 0.006	− 0.005		
		(0.006)	(0.007)		
Level 3		− 0.015**	− 0.011		
		(0.006)	(0.008)		
Level 4		− 0.026***	− 0.038***		
		(0.007)	(0.009)		
Level 5		− 0.027***	− 0.040***		
		(0.009)	(0.011)		
Social relationships^a^					
Level 2		− 0.015***	− 0.080***		
		(0.004)	(0.005)		
Level 3		− 0.035***	− 0.150***		
		(0.006)	(0.008)		
Level 4		− 0.076***	− 0.211***		
		(0.009)	(0.011)		
Level 5		− 0.082***	− 0.217***		
		(0.014)	(0.018)		
Community connectedness^a^					
Level 2		− 0.006	− 0.017***		
		(0.005)	(0.006)		
Level 3		− 0.012**	− 0.067***		
		(0.005)	(0.007)		
Level 4		− 0.020**	− 0.080***		
		(0.008)	(0.010)		
Level 5		− 0.026**	− 0.116***		
		(0.011)	(0.014)		
Observations	7846	7846	8005	3812	3809
*R*^2^	0.434	0.503	0.447	0.371	0.404

Standard errors in parentheses

*MIC* multi-instrument-comparison; *NHMS* National Health Measurement Study; *SWLS* satisfaction with life scale; *VAS* visual analogue scale. The SWLS was calculated as a summary score of the first three items of the SWLS instrument and then rescaled onto the 0–1 scale. The VAS was originally recorded on a 0–100 scale and was rescaled onto 0–1 scale

***p < 0.01, **p < 0.05, *p < 0.1

^a^Bolt-on dimensions came from AQoL-8D in MIC data

^b^Bolt-on dimensions came from SF-6D in NHMS data. All regression also includes a constant. The EQ-5D-5L was used in the MIC data, while EQ-5D-3L was used in the NHMS data

Table 3 presents detailed regression results for all five EQ-5D dimensions (Column 1), as well as further including the four bolt-on dimensions (Column 2). First, the inclusion of four bolt-on dimensions increased the *R*^2^ from 0.434 to 0.503 (adjusted *R*^2^ increased from 0.433 to 0.501). Second, estimated coefficients for the four bolt-on dimensions were statistically significant in the expected sign (with exception of the Level 2 of the *Sleep* and *Community Connectedness* dimensions which were indifferent with Level 1). These four bolt-on dimensions had very limited impacts on the estimated coefficients of the original EQ-5D dimensions. Column 3 of the table used SWLS as the dependent variable. The estimated *R*^2^ was 0.447. The *Anxiety/Depression* as well as four bolt-ons were statistically significant, while the other four original EQ-5D dimensions were mostly insignificant.

To facilitate a clearer view on the relative importance of HRQoL dimensions on VAS and SWLS, Table 4 Columns 1–3 reported the corresponding Shorrocks-Shapely decomposition of the *R*^2^ for each column in Table 3. Among all four bolt-ons, it was evident that *Vitality* stood out as the most important dimension in explaining the variance of VAS. The other three bolt-on dimensions were also consistently more important than the *Self-Care* dimension in the EQ-5D. Column 3, as a comparison, shows that when explaining the variance of SWLS, *Social Relationships* was the most important dimension, followed by *Anxiety/Depression*, *Community Connectedness,* and *Vitality*. The remaining four EQ-5D dimensions played the least important roles.

**Table 4:** Relative contribution of EQ-5D and bolt-on dimension(s) on VAS or SWLS (%)

	MIC data			NHMS data	
	(1)	(2)	(3)	(4)	(5)
	VAS	VAS	SWLS	EQ-VAS	EQ-VAS
Mobility	16.8	10.7	2.1	20.8	16.0
Self-care	8.7	5.4	1.6	10.9	8.5
Usual activities	25.9	15.1	4.7	34.1	25.8
Pain/discomfort	19.9	11.7	3.2	22.4	17.5
Anxiety/depression	28.7	11.7	23.2	11.8	8.4
Vitality		23.0	16.4		23.7
Sleep		7.8	7.9		
Social relationships		7.3	24.0		
Community connectedness		7.4	16.9		

The Shorrocks-Shapely decomposition of *R*^2^ reported here correspond to the OLS estimates in Table 3

*MIC* multi-instrument-comparison; *NHMS* National Health Measurement Study; *SWLS* satisfaction with life scale; *VAS* visual analogue scale

Given that the MIC data include multiple self-reported chronic diagnosis groups, as well as a non-diagnosed group, referred to as the healthy group, we further presented the Shorrocks-Shapely decomposition results for three selected sub-samples. We chose (i) the healthy group, because it is the only group of respondents who reported no specific diagnosis; (ii) the group reporting ever been diagnosed with arthritis, because it was considered the most somatic of the diagnosis groups in MIC, and (iii) the group reporting ever been diagnosed with depression, because it was the only group reporting a diagnosis of mental illness.

**Fig. 2:**
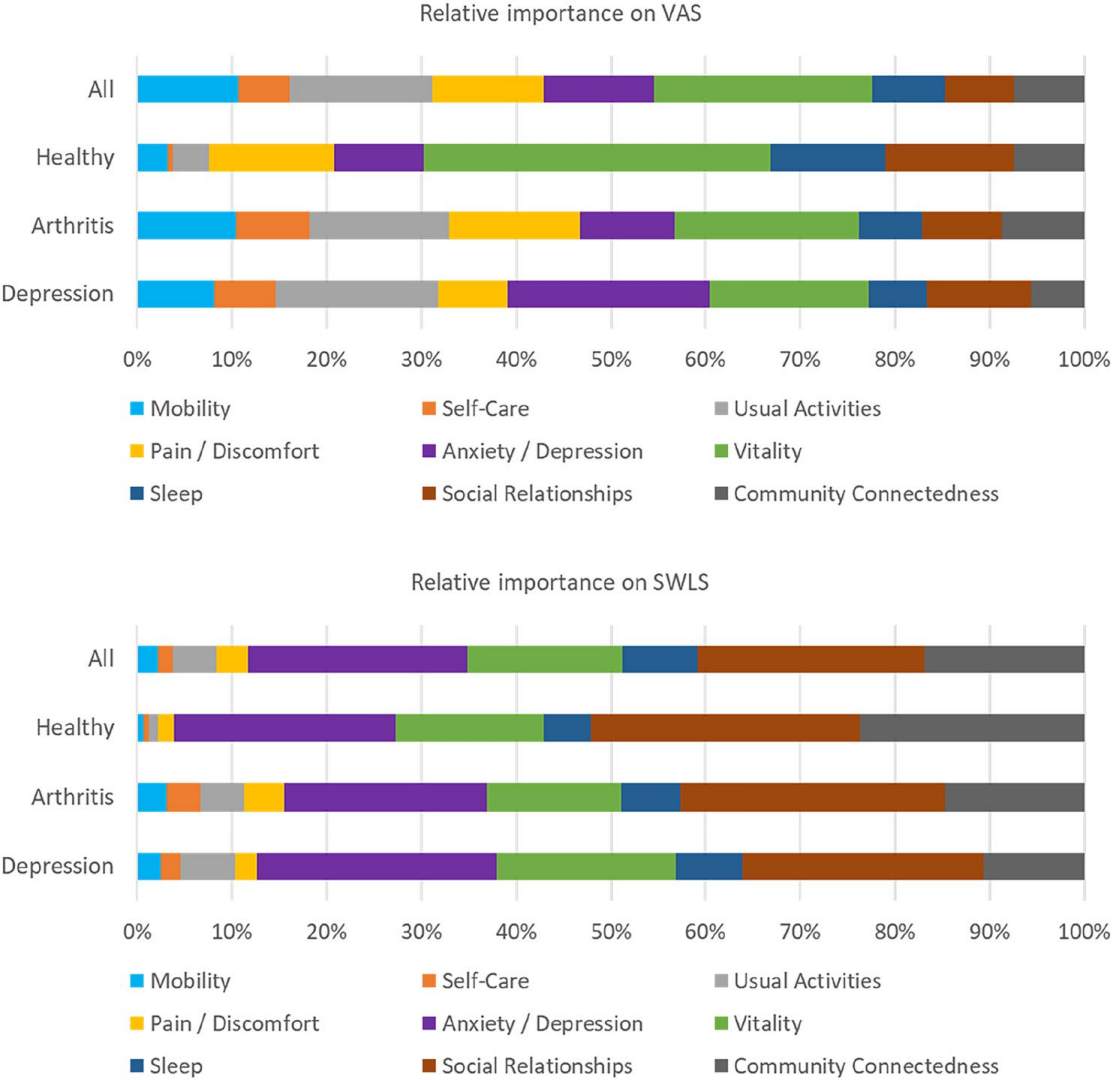
Shorrocks-Shapely decomposition results by three diagnostic groups (healthy, arthritis, depression) and two outcome measures (VAS and SWLS)

Figure 2 shows how the relative importance of the nine dimensions varied by disease status. First, to explain VAS, for the healthy group, *Vitality* was the most important dimension (36.6%), followed by *Social Relationships* (13.6%), *Pain/Discomfort* (13.3%), and *Sleep* (12.1%); *Self-Care* was the least important (0.6%). For the arthritis group, *Vitality* again was the most important dimension (19.5%), followed by *Usual Activities* (14.8%), *Pain/Discomfort* (13.8%), and *Mobility* (10.5%); *Sleep* was the least important (6.7%). Lastly, for the depression group, *Anxiety/Depression* was the most important (21.3%), followed by *Usual Activities* (17.1%), *Vitality* (16.8%), and *Social Relationships* (11.1%); *Community Connectedness* was the least important (5.6%). Second, when investigating the importance of these nine dimensions on explaining SWLS, it can be seen from Fig. 2 that *Social Relationships, Anxiety/Depression*, *Vitality*, *Community Connectedness*, and *Sleep* played more important roles.

The latent structure of the EQ-5D-5L dimensions as well as four bolt-on dimensions are demonstrated using EFA in Table 5. Two factors were extracted. Except for the *Anxiety/Depression*, all other four EQ-5D dimensions were grouped together to represent physical health. The four bolt-on dimensions along with the *Anxiety/Depression* were grouped together to represent psycho-social health.

**Table 5:** Exploratory factor analysis

	Factor	
	1	2
[EQ-5D] Mobility	0.924	
[EQ-5D] Usual activities	0.803	
[EQ-5D] Pain/discomfort	0.654	
[EQ-5D] Self-care	0.614	
[EQ-5D] Anxiety/depression		0.768
[Bolt-on] Community connectedness		0.726
[Bolt-on] Social relationships		0.700
[Bolt-on] Vitality		0.561
[Bolt-on] Sleep		0.510

Pattern matrix presented. Extraction method: maximum likelihood. Number of factors was determined by the minimum average partial method. Rotation Method: Oblique Promax (Kappa = 4) with Kaiser Normalization. Rotated factor correlation: 0.57. Loadings smaller than 0.3 are not shown in the table

### Robustness analysis

The *Vitality* dimension stood out with such importance that we inquired into an alternative data source collected in the USA in support [12]. Interestingly, the alternative data confirmed the important relative position of *Vitality* (which was now measured based on the item used in SF-6D [19]). The Shorrocks-Shapely decomposition result found that *Vitality* was the second most important dimension (23.7%) in explaining VAS, following *Usual Activities* (25.8%) (Tables 2 and 3, Columns 4–5).

### Mapping analysis

In the Electronic Supplementary Material 2, we documented the process and results from a direct mapping analysis to link the EQ-5D-5L and four bolt-on dimensions onto the AQoL-8D utility scores and the final mapping function is presented in Table 6. All nine dimensions were statistically significant in predicting AQoL-8D utility score. In total, these nine dimensions explained around 90% variance of the AQoL-8D.

**Table 6:** Mapping EQ-5D + 4 bolt-on dimensions onto AQoL-8D utility scores

Dimensions	Levels	Coefficient	SE
Mobility	2	− 0.0154***	(0.003)
	3	− 0.0215***	(0.004)
	4 & 5	− 0.0323***	(0.006)
Self-care	2	− 0.0262***	(0.004)
	3, 4 & 5	− 0.0359***	(0.005)
Usual activities	2	− 0.0251***	(0.003)
	3	− 0.0404***	(0.004)
	4	− 0.0438***	(0.007)
	5	− 0.0589***	(0.012)
Pain/discomfort	2	− 0.0270***	(0.002)
	3	− 0.0623***	(0.003)
	4	− 0.1107***	(0.004)
	5	− 0.1286***	(0.008)
Anxiety/depression	2	− 0.0652***	(0.002)
	3	− 0.1292***	(0.003)
	4	− 0.1711***	(0.005)
	5	− 0.1902***	(0.007)
Vitality	2	− 0.0216***	(0.003)
	3	− 0.0862***	(0.003)
	4	− 0.1472***	(0.004)
	5	− 0.1774***	(0.005)
Sleep	2	− 0.0230***	(0.003)
	3	− 0.0502***	(0.003)
	4	− 0.0708***	(0.003)
	5	− 0.0738***	(0.004)
Social relationships	2	− 0.0450***	(0.002)
	3	− 0.0954***	(0.003)
	4	− 0.1101***	(0.004)
	5	− 0.1114***	(0.007)
Community connectedness	2	− 0.0405***	(0.002)
	3	− 0.1072***	(0.003)
	4	− 0.1332***	(0.004)
	5	− 0.1456***	(0.006)
Constant		1	

Standard errors (SE) in parentheses

***p < 0.01, **p < 0.05, *p < 0.1. Dependent variable: AQoL-8D utility. The first level of each dimension was the omitted level. The constant was constrained to be 1. See Electronic Supplementary Table 4 for more details

Based on the reported coefficients, the health utility score of a particular health state defined according to these nine dimensions can be calculated. Let a single-digit number (ranges from 1 to 5) indicate the response level of a dimension, a health state can be described using a 9-digit code, e.g. the full health can be described as ‘111,111,111′. Take a health state of ‘123,451,234′ as an example, the health utility score can be calculated as follows:

$$ \begin{aligned} {\text{Health utility}}_{{({123451234})}} & = { 1 }{-} \, 0 \, {-} \, 0.0{262 }{-} \, 0.0{4}0{4 }{-} \, 0.{11}0{7} \\ & \quad {-} \, 0.{19}0{2 }{-} \, 0 \, {-} \, 0.0{23}0 \, {-} \, 0.0{954 }{-} \, 0.{1332 } = \, 0.{38}0{9}. \\ \end{aligned} $$

## Discussion

Given the dominant position of the EQ-5D in applied studies, we believe that identifying a set of bolt-on dimensions that capture psycho-social aspects of health would serve as a realistic alternative (at least in the short run) for developing a completely new extended GPBM. Based on the world’s largest relevant data set, we tested the empirical support for four bolt-on dimensions. In total, they explain 45% of the *R*^2^ in the HRQoL equation (as measured by VAS) and 65% of the R^2^ in the GLS equation (as measured by SWLS). Among the four, *Vitality* was most important for HRQoL (23%), while *Social Relationships* was most important for GLS (24%).

These results are supportive of the findings in recent papers by Finch et al. [20, 21] that were based on the same MIC data. However, their analytical approach was different from ours, in that they included ‘satisfaction’, as measured by a wide range of life satisfaction and subjective well-being dimensions including the SWLS dimensions, to explore their ability to predict HRQoL [21]. In our analysis, SWLS was considered a separate *dependent* variable, rather than an *independent* variable for HRQoL. Thereby, the analytical framework we adopted here could directly explore any differences in the relative importance of the nine dimensions (EQ-5D plus four bolt-on dimensions) to predict HRQoL, as compared to GLS. Mukuria and Brazier [22] empirically explored weighting EQ-5D-3L or SF-6D dimensions against an overall (5 level) happiness indicator. They found that mental health, vitality, and social functioning had a stronger association with respondents’ happiness, while the pain had a weak association, and physical health had no association. The results from our study are broadly supportive to their findings. However, distinctions also exist. First, we further quantify the contribution of each dimension clearly by using the Shorrocks-Shapely decomposition analysis. Second, we are able to compare the relative importance when weighting against either health or life satisfaction directly under the identical analysis framework. Last but not the least, this paper investigated the dimensions following a theoretical framework been proposed by O&M [5] and with an aim to propose a feasible solution to fill the psycho-social gap in the EQ-5D.

The proposed psycho-social bolt-on dimensions to the EQ-5D health state classification system also contributes to the current discussion of using subjective well-being as compared to health state utility in economic evaluation, i.e. whether they are complements or substitutes. The majority of empirical evidence found that GPBM (e.g. EQ-5D) and subjective well-being are complementary measures: such as the EQ-5D-3L versus the ONS4 (Office for National Statistics 4) and the subjective well-being questions in Parkinson’s disease patients [23]; EQ-5D-5L versus WHO-5 well-being index in psoriasis vulgaris patients [24], and EQ-5D-5L versus multiple subjective well-being measures in patients with heart diseases [25]. A key reason is owing to the EQ-5D health state classification system [11]: When a GPBM with a broader psycho-social dimensions (e.g. AQoL-8D) been used as a comparator, the additional information provided by a subjective well-being instrument was substantially reduced [26]. The EQ-5D and four proposed bolt-on dimensions may partially provide a solution to produce a brief instrument that capture a broader notion of ‘health and well-being’. Outside the preference-based HRQoL, such exploration on creating an overarching framework for quality of life and subjective well-being, see Skevington and Böhnke [27].

The comprehensiveness of a health state classification system does not come without limitation. In particular, the feasibility to be widely used in clinical trials and large-scale population survey could be impacted. The EQ-5D-5L along with the four new psycho-social bolts could help fill in the gap. In Electronic Supplementary Material 2, we compared the strength of correlations between EQ-5D-5L utility, the bolt-on version EQ-5D-5L scored using the mapping algorithm (reported in Table 6), or AQoL-8D and two mental health-specific instruments based on a sub-sample of 917 respondents with depression. As shown in Supplementary Table 5, a clear improvement on the strength of associations with the bolt-on version, as compared to the EQ-5D-5L can be found (although the magnitudes of the correlation coefficients were still smaller than AQoL-8D). This preliminary analysis suggests that with the additional four bolt-on dimensions, the performance of EQ-5D-5L on psycho-social health can be evidently improved.

There are several caveats of this study. First, there are multiple ways to select bolt-on dimensions for EQ-5D. This study does not aim to empirically explore the potential bolt-on dimensions from scratch, but instead start from the theoretical framework outlined in the O&M paper [5]. Readers who are interested to know more about developing EQ-5D bolt-on dimensions, see Longworth et al. [3]. Second, the four bolt-on dimensions were directly drawn from an existing GPBM, i.e. the AQoL-8D. The wordings for each item as well as its response levels were pre-defined according the AQoL-8D classification system, which differs from the EQ-5D classification system. For further development of the proposed psycho-social bolt-ons, it is important to describe the levels in a coherent way to better align with the standard EQ-5D classification system. In the Electronic Supplementary Material 3, we have included a suggested description of the four bolt-ons’ five levels by use of the EQ nomenclature. Certainly, more work is needed on developing an EQ-adapted description of these four dimensions, something that we believe would best be taken care of by the EuroQol Group.

Third, although this paper briefly demonstrated the potential performance of EQ-5D and four bolt-on dimensions in the Supplementary document, the valuation study should be conducted from representative general population to develop the value set for this new health state classification system. The current paper focused on the importance of these added dimensions in explaining health state values (VAS) and global life satisfaction among different groups of respondents, most of whom with chronic conditions, i.e. they signalled *experienced* preferences. A next phase for research is to include the bolt-on dimensions together with the EQ-5D dimensions in a coherent descriptive system in order to undertake a valuation study, using time-trade-off (TTO) or discrete choice experiments (DCE), with the aim of eliciting preference weights among respondents placed in the role of imagining themselves in the *hypothetical* health states [28–30]. It remains to be seen whether the relative importance of these bolt-on dimensions, reported here, will differ when asking people in their role of hypothetical patients.

Fourth, the MIC data reflect preferences among respondents in six relatively rich countries (Australia, Canada, Germany, Norway, UK, US). Whether the conclusion from this study is also applicable to developing countries should be further investigated.

## Conclusion

Since 1990 when the EQ-5D was first introduced, the disease patterns in these countries have changed and with more open public concerns on the psycho-social aspects of health. This might help explain the significant importance assigned to *Vitality, Sleep, Social relationships*, and *Community connectedness*. More work is needed on how to phrase the four bolt-on dimensions in order to make them appear like a stand-alone supplement with a coherent descriptive structure.

## Electronic supplementary material

Below is the link to the electronic supplementary material.

Supplementary file 1 (DOCX 506 kb)
