# Supplementary material for: Filling the psycho-social gap in the EQ-5D: the empirical support for four bolt-on dimensions
Source: Qual Life Res. 2020 Jul 9;29(11):3119–29. doi: 10.1007/s11136-020-02576-5 (PMC7591404; doi:10.1007/s11136-020-02576-5)
Supplement: Supplementary file 1 — (DOCX 506 kb) [file 11136_2020_2576_MOESM1_ESM.docx]

# **Filling the psycho-social gap in the EQ-5D: The empirical support for four bolt-on dimensions**

Gang Chen^1^, Jan Abel Olsen^2, 3*^

1 Centre for Health Economics, Monash Business School, Monash University, Victoria 3145, Australia

2 Department of Community Medicine, University of Tromsø, 9037 Tromsø, Norway

3 Division of Health Services, Norwegian Institute of Public Health, 0213 Oslo, Norway

ORCID:

Chen G: 0000-0002-8385-5965

Olsen JA: 0000-0001-9472-2669

^*^ Corresponding author:

Professor Jan Abel Olsen, Department of Community Medicine, University of Tromsø, NO 9037 Tromsø,

Tel: +47 4133 2054

Email: [jan.abel.olsen@uit.no](mailto:jan.abel.olsen@uit.no)

**Electronic Supplementary Material 1**

| **Supplementary Table 1** Descriptive statistics of respondents in Multi-Instrument-Comparison (MIC) study, % | | | | | | | | | | | |
| --- | --- | --- | --- | --- | --- | --- | --- | --- | --- | --- | --- |
|  | ALL | |  | Healthy | |  | Arthritis | |  | Depression | |
|  | VAS equation | SWLS equation |  | VAS equation | SWLS equation |  | VAS equation | SWLS equation |  | VAS equation | SWLS equation |
|  | N=7,846 | N=8,005 |  | N=1,587 | N=1,760 |  | N=929 | N=926 |  | N=917 | N=913 |
| Gender |  |  |  |  |  |  |  |  |  |  |  |
| Female | 52.1 | 52.0 |  | 52.6 | 52.2 |  | 63.7 | 63.9 |  | 65.9 | 65.7 |
| Age (years) |  |  |  |  |  |  |  |  |  |  |  |
| 18-34 | 18.0 | 18.1 |  | 28.5 | 28.5 |  | 6.1 | 6.1 |  | 35.2 | 35.1 |
| 35-54 | 35.3 | 35.3 |  | 38.2 | 37.7 |  | 37.1 | 37.3 |  | 47.6 | 47.7 |
| 55-64 | 25.2 | 25.0 |  | 16.1 | 15.9 |  | 32.9 | 32.9 |  | 13.3 | 13.4 |
| 65+ | 21.5 | 21.6 |  | 17.2 | 17.8 |  | 23.8 | 23.8 |  | 3.9 | 3.9 |
| Education |  |  |  |  |  |  |  |  |  |  |  |
| University degree | 28.0 | 28.2 |  | 26.8 | 27.6 |  | 24.4 | 24.4 |  | 25.9 | 25.9 |

| **Supplementary Table 2** Descriptive statistics for EQ-5D-5L, % | | | |
| --- | --- | --- | --- |
|  |  | Panel A | Panel B |
|  |  | VAS equation | SWLS equation |
| EQ-5D-5L Dimensions | Levels | N=7,846 | N=8,005 |
| MOBILITY | 1 | 66.1 | 66.5 |
|  | 2 | 18.7 | 18.6 |
|  | 3 | 10.5 | 10.3 |
|  | 4 | 4.3 | 4.3 |
|  | 5 | 0.4 | 0.4 |
| SELF-CARE | 1 | 87.5 | 87.7 |
|  | 2 | 8.2 | 8.1 |
|  | 3 | 3.5 | 3.4 |
|  | 4 | 0.8 | 0.8 |
|  | 5 | 0.1 | 0.1 |
| USUAL ACTIVITIES | 1 | 64.0 | 64.6 |
|  | 2 | 22.0 | 21.7 |
|  | 3 | 10.1 | 9.9 |
|  | 4 | 3.3 | 3.2 |
|  | 5 | 0.7 | 0.6 |
| PAIN / DISCOMFORT | 1 | 28.6 | 29.2 |
|  | 2 | 40.6 | 40.5 |
|  | 3 | 20.5 | 20.1 |
|  | 4 | 8.9 | 8.7 |
|  | 5 | 1.5 | 1.4 |
| ANXIETY / DEPRESSION | 1 | 49.6 | 50.0 |
|  | 2 | 29.3 | 29.3 |
|  | 3 | 14.0 | 13.8 |
|  | 4 | 5.0 | 4.9 |
|  | 5 | 2.1 | 2.0 |
| SWLS, Satisfaction With Life Scale; VAS, Visual Analogue Scale. | | | |


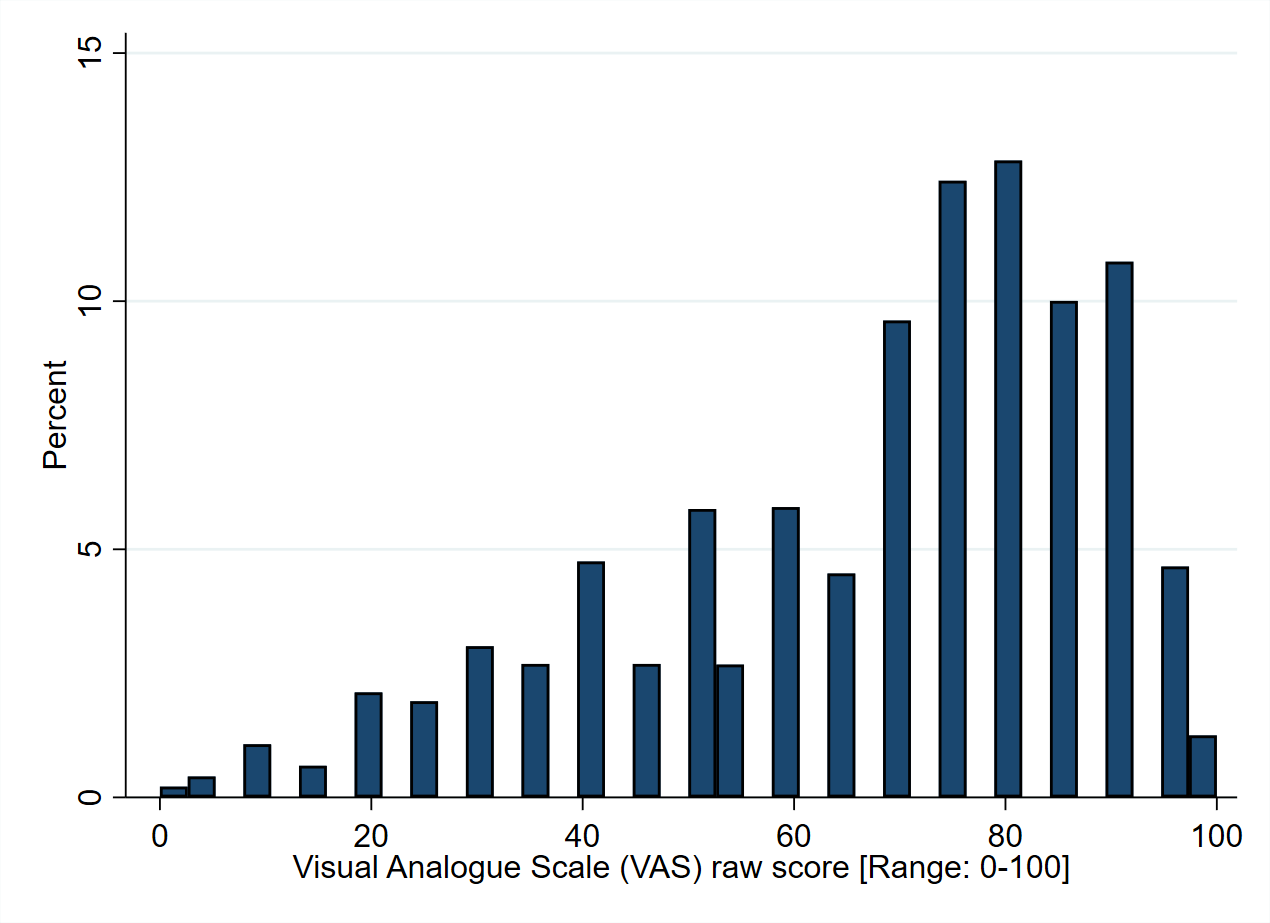


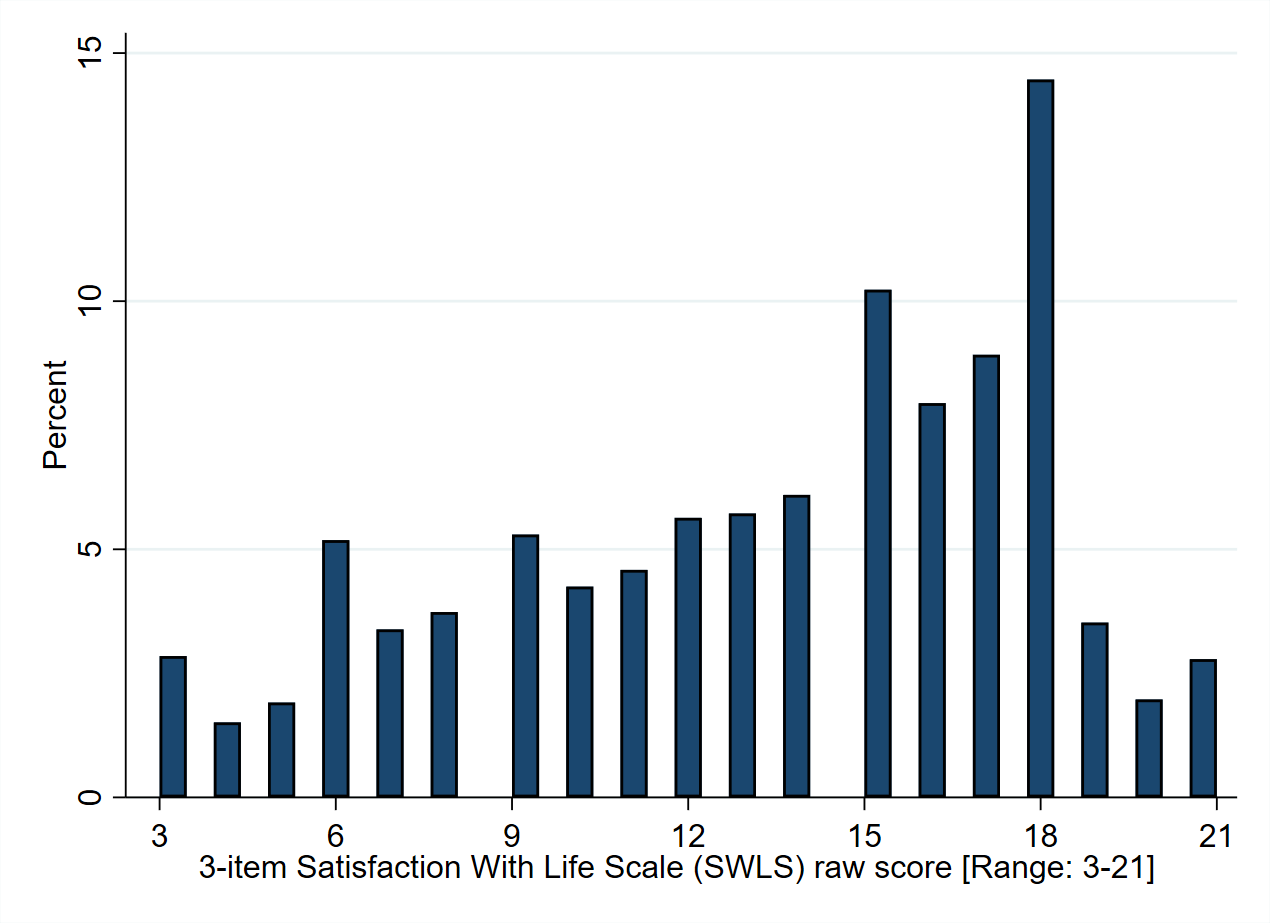


**Supplementary Figure 1** Histograms of the VAS and SWLS raw scores

| **Supplementary Table 3** R^2^ statistics when one dimension was included as a time | | |
| --- | --- | --- |
|  | Panel A | Panel B |
|  | VAS equation | SWLS equation |
| Dimensions | N=7,846 | N=8,005 |
| Mobility | 0.24 | 0.07 |
| Self-Care | 0.15 | 0.05 |
| Usual Activities | 0.32 | 0.12 |
| Pain / Discomfort | 0.26 | 0.09 |
| Anxiety / Depression | 0.24 | 0.31 |
| Vitality | 0.37 | 0.25 |
| Sleep | 0.20 | 0.16 |
| Social Relationships | 0.16 | 0.28 |
| Community Connectedness | 0.19 | 0.26 |

**Electronic Supplementary Material 2**

By focusing on six generic preference-based measures (15D, AQoL-8D, EQ-5D-5L, HUI3, QWB, SF-6D), Richardson et al. [2015] shown that “to a large extent, utility can account for variation in subjective wellbeing” and among these instruments, the EQ-5D-5L takes least account whilst AQoL-8D most account of subjective wellbeing. The key reason is the wide variations in which dimensions that are included in the descriptive systems. Meanwhile, a potential barrier for AQoL-8D to be widely used lies in its classification system, which consists of a total of 35 items.

By conducting a direct mapping analysis using the ordinary least squares estimator, we explored to what extent the EQ-5D-5L and four bolt-on dimensions (i.e. a total of 9 items) can explain the variation of AQoL-8D utility scores. Here, we regress the AQoL-8D utility scores on EQ-5D-5L dimensions and further including the four bolt-on dimensions using data from 8,019 respondents who answered both two instruments. The nine dimensions were included in the regression analysis as a series of dummy variables to allow for the potential non-linear effect. Since the key purpose is to demonstrate the potential performance of the new health classification system, we did not explore other potential econometric methods. So, the mapping algorithm we developed here may not be the optimal one. More details on mapping EQ-5D-5L onto AQoL-8D utility scores can be found at Chen et al. [2016].

In Supplementary Table 4, we first include EQ-5D-5L dimensions only, and they explained 73% variations of the AQoL-8D utility score. Next, when the four bolt-on dimensions were further included, all nine dimensions explained 89% variations of the AQoL-8D. All estimation shown expected sign as well as the relative magnitude of coefficients within each dimension, with three exceptions: For *Mobility* dimension, the most severe levels (e.g. Level 5) was insignificant, whilst for *Self-care* dimension, the magnitude of the estimated coefficient for Level 4 was smaller than Level 3, and the estimated Level 5 was insignificant. Following the commonly adopted procedure in health state valuation study, for the final mapping algorithm presented in Table 6, the inconsistent levels were combined together. In addition, since the estimated coefficient for constant was 0.9909, for the ease of using the mapping algorithm, we have also constrained it to be 1.0 in the final mapping function. In this case, if respondents score Level 1 in all 9 dimensions (i.e. full health), the predicted utility equals to 1.0.

The mean absolute error (MAE) of the final mapping function reported in Table 6 was 0.058. The scatter plot between the observed AQoL-8D utility score and the predicted scores is presented in Supplementary Figure 2, and the distribution of the prediction error is shown in Supplementary Figure 3. A simple internal validation analysis was conducted in which the final mapping was tested on two random samples with sample sizes of 1,000 and 5,000 respectively which were generated by a random selection within the full sample (see Validation II in Chen et al. [2014]). The MAEs of this internal validation analysis were 0.059 and 0.058, respectively. Based on above evidence, the reported mapping algorithm has good mapping performance. Further external validation analysis should be conducted if external data become available.

To further demonstrate the performance of these nine dimensions scored by using the mapping algorithm, we calculate the Spearman’s correlation coefficients between each of EQ-5D-5L; EQ-5D-5L plus the four bolt-on dimensions, and; AQoL-8D utility, with two mental health specific instruments: the Depression Anxiety Stress Scales (DASS-21) and the K10 (for more details on the instruments and their associations between generic preference based measures, see Mihalopoulos et al. [2014]). As can be seen in Supplementary Table 5, for a sub-sample of 917 depression respondents who reported all required instruments, adding bolt-on dimensions had led to a clear improvement on the magnitude of correlations with the two disease-specific instruments.

| **Supplementary Table 4** Mapping EQ-5D & bolt-on dimensions onto AQoL-8D utility scores | | | | | | |
| --- | --- | --- | --- | --- | --- | --- |
|  |  | EQ-5D dimensions | |  | EQ-5D + 4 bolt-ons | |
| Dimensions | Levels | Coefficient | SE |  | Coefficient | SE |
| MOBILITY | 2 | -0.0258*** | (0.004) |  | -0.0154*** | (0.003) |
|  | 3 | -0.0352*** | (0.006) |  | -0.0213*** | (0.004) |
|  | 4 | -0.0520*** | (0.009) |  | -0.0342*** | (0.006) |
|  | 5 | -0.0545** | (0.023) |  | -0.0109 | (0.015) |
| SELF-CARE | 2 | -0.0377*** | (0.005) |  | -0.0263*** | (0.004) |
|  | 3 | -0.0429*** | (0.009) |  | -0.0388*** | (0.006) |
|  | 4 | -0.0376** | (0.017) |  | -0.0245** | (0.011) |
|  | 5 | -0.0357 | (0.043) |  | -0.0350 | (0.028) |
| USUAL ACTIVITIES | 2 | -0.0668*** | (0.004) |  | -0.0252*** | (0.003) |
|  | 3 | -0.1017*** | (0.006) |  | -0.0398*** | (0.004) |
|  | 4 | -0.1165*** | (0.010) |  | -0.0441*** | (0.007) |
|  | 5 | -0.1368*** | (0.019) |  | -0.0627*** | (0.012) |
| PAIN / DISCOMFORT | 2 | -0.0446*** | (0.003) |  | -0.0264*** | (0.002) |
|  | 3 | -0.0998*** | (0.005) |  | -0.0618*** | (0.003) |
|  | 4 | -0.1477*** | (0.006) |  | -0.1102*** | (0.004) |
|  | 5 | -0.1672*** | (0.013) |  | -0.1292*** | (0.008) |
| ANXIETY / DEPRESSION | 2 | -0.1491*** | (0.003) |  | -0.0652*** | (0.002) |
|  | 3 | -0.2871*** | (0.004) |  | -0.1291*** | (0.003) |
|  | 4 | -0.3725*** | (0.007) |  | -0.1715*** | (0.005) |
|  | 5 | -0.4217*** | (0.010) |  | -0.1897*** | (0.007) |
| VITALITY | 2 |  |  |  | -0.0146*** | (0.004) |
|  | 3 |  |  |  | -0.0795*** | (0.004) |
|  | 4 |  |  |  | -0.1406*** | (0.005) |
|  | 5 |  |  |  | -0.1709*** | (0.006) |
| SLEEP | 2 |  |  |  | -0.0211*** | (0.003) |
|  | 3 |  |  |  | -0.0485*** | (0.003) |
|  | 4 |  |  |  | -0.0692*** | (0.004) |
|  | 5 |  |  |  | -0.0721*** | (0.004) |
| SOCIAL RELATIONSHIPS | 2 |  |  |  | -0.0447*** | (0.002) |
|  | 3 |  |  |  | -0.0951*** | (0.003) |
|  | 4 |  |  |  | -0.1098*** | (0.004) |
|  | 5 |  |  |  | -0.1109*** | (0.007) |
| COMMUNITY CONNECTEDNESS | 2 |  |  |  | -0.0402*** | (0.002) |
|  | 3 |  |  |  | -0.1070*** | (0.003) |
|  | 4 |  |  |  | -0.1331*** | (0.004) |
|  | 5 |  |  |  | -0.1456*** | (0.006) |
| Constant |  | 0.8889*** | (0.003) |  | 0.9909*** | (0.004) |
| Observations |  | 8,019 |  |  | 8,019 |  |
| R-squared |  | 0.73 |  |  | 0.89 |  |
| Standard errors in parentheses. *** p<0.01, ** p<0.05, * p<0.1. Dependent variable: AQoL-8D utility. The first level of each dimension was the omitted level. | | | | | | |


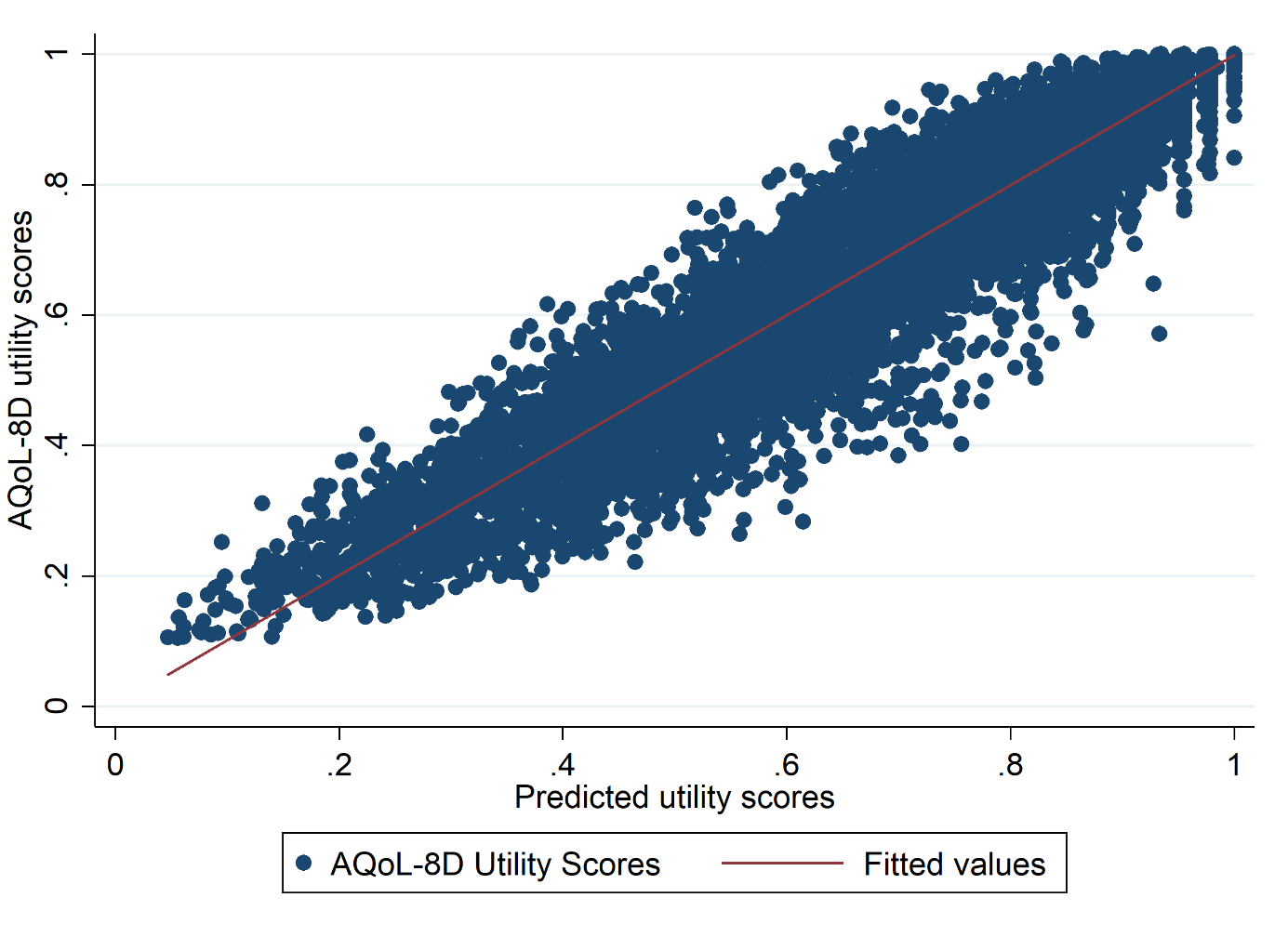


**Supplementary Figure 2** Scatter plots of the observed versus predicted utility scores


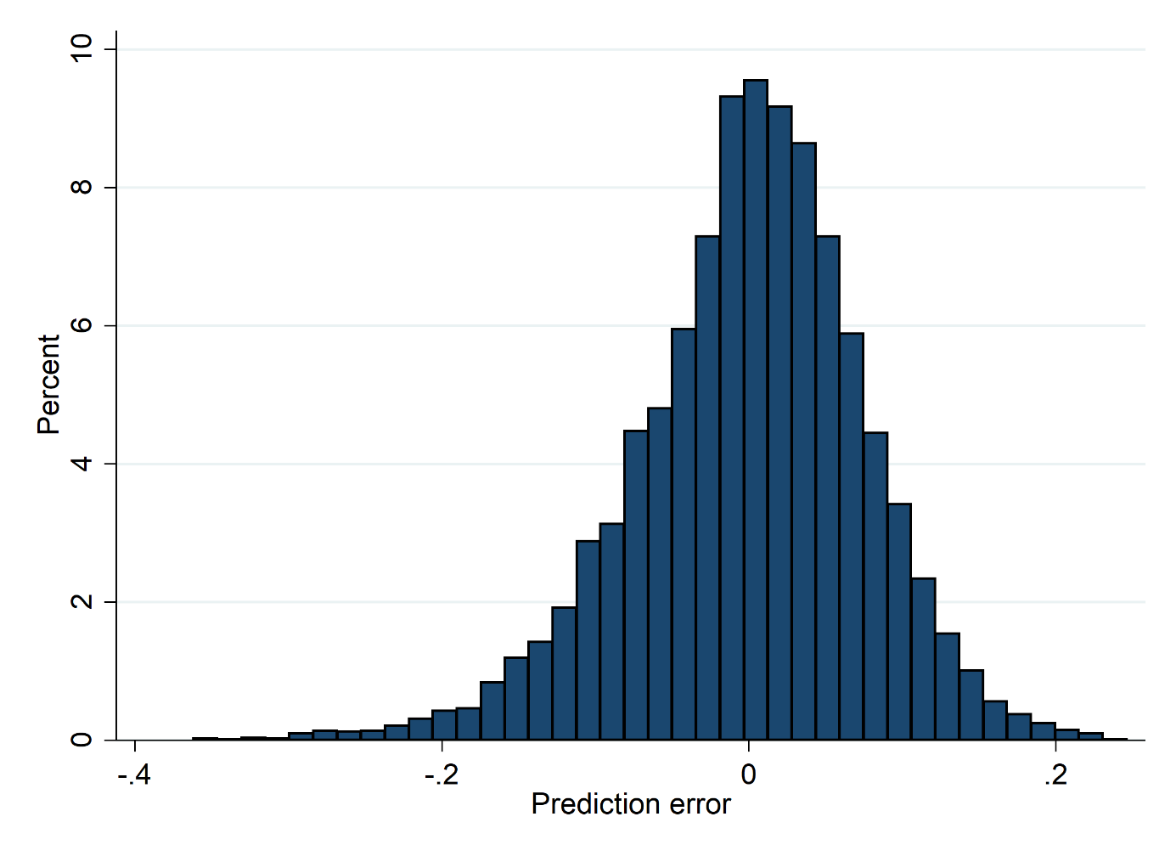


**Supplementary Figure 3** Distribution of the mapping prediction error

| **Supplementary Table 5** Spearman's correlation coefficients between three generic and disease-specific instruments (N=917) | | | |
| --- | --- | --- | --- |
|  | EQ-5D-5L | EQ-5D-5L + 4 bolt-ons | AQoL-8D |
| DASS-21 |  |  |  |
| Stress | 0.438 | 0.503 | 0.532 |
| Depression | 0.512 | 0.625 | 0.702 |
| Anxiety | 0.501 | 0.530 | 0.570 |
| K10 | 0.572 | 0.654 | 0.731 |
| All correlation coefficients were statistically significant (P<0.001). | | | |

## References

Richardson, J., Chen, G., Khan, M.A., & Iezzi, A. (2015). Can Multi-Attribute Utility Instruments Adequately Account for Subjective Well-Being? *Medical Decision Making*, *35*(3):292-304.

Chen, G., Khan, M.A., Iezzi, A., Ratcliffe, J., & Richardson, J. (2016). Mapping between 6 Multiattribute Utility Instruments. *Medical Decision Making*, *36*(2):160-175.

Chen, G., Stevens, K., Rowen, D., Ratcliffe, J. (2014). From KIDSCREEN-10 to CHU9D: Creating a Unique Mapping Algorithm for Application in Economic Evaluation. *Health and Quality of Life Outcomes*, 12: 134.

Mihalopoulos, C., Chen, G., Iezzi, A., Khan, M.A., & Richardson, J. (2014). Assessing Outcomes for Cost-Utility Analysis in Depression: Comparison of Five Multi-Attribute Utility Instruments with Two Depression-Specific Outcome Measures. *British Journal of Psychiatry*, *205*(5):390-397.

**Electronic Supplementary Material 3**

An alternative description of the four bolt-ons, using the EQ-nomenclature

(Draft)

**Vitality** (feeling energetic)

- I have no problems with lack of energy
- I have slight problems with lack of energy
- I have moderate problems with lack of energy
- I have severe problems with lack of energy
- I have extreme problems with lack of energy

**Sleep**

- I have no problems with sleeping
- I have slight problems with sleeping
- I have moderate problems with sleeping
- I have severe problems with sleeping
- I have extreme problems with sleeping

**Personal relationships** (family and friends)

- I have no problems with my personal relationships
- I have slight problems with my personal relationships
- I have moderate problems with my personal relationships
- I have severe problems with my personal relationships
- I have extreme problems with my personal relationships

**Social isolation** (feeling part of community)

- I have no problems with social isolation
- I have slight problems with social isolation
- I have moderate problems with social isolation
- I have severe problems with social isolation
- I have extreme problems with social isolation
